# Animal Models of the Neuromuscular Junction, Vitally Informative for Understanding Function and the Molecular Mechanisms of Congenital Myasthenic Syndromes

**DOI:** 10.3390/ijms19051326

**Published:** 2018-04-29

**Authors:** Richard G. Webster

**Affiliations:** Nuffield Department of Clinical Neurosciences, University of Oxford, Oxford OX3 9DS, UK; richard.webster@ndcn.ox.ac.uk

**Keywords:** neuromuscular junction, animal model, myasthenia, congenital

## Abstract

The neuromuscular junction is the point of contact between motor nerve and skeletal muscle, its vital role in muscle function is reliant on the precise location and function of many proteins. Congenital myasthenic syndromes (CMS) are a heterogeneous group of disorders of neuromuscular transmission with 30 or more implicated proteins. The use of animal models has been instrumental in determining the specific role of many CMS-related proteins. The mouse neuromuscular junction (NMJ) has been extensively studied in animal models of CMS due to its amenability for detailed electrophysiological and histological investigations and relative similarity to human NMJ. As well as their use to determine the precise molecular mechanisms of CMS variants, where an animal model accurately reflects the human phenotype they become useful tools for study of therapeutic interventions. Many of the animal models that have been important in deconvolving the complexities of neuromuscular transmission and revealing the molecular mechanisms of disease are highlighted.

## 1. Introduction

Communication of the motor nerve signal with the muscle fibre that leads to controlled muscle contraction is a vital physiological function. The neuromuscular junction (NMJ), the point of contact between nerve and muscle, is a specialised structure that ensures this communication can occur reliably and repeatedly, even under extreme conditions, without fail. Disorder of this structure, through defects in its formation, maintenance or malfunction of its elements, can lead to communication failure and pathophysiology.

Neurotransmission is achieved by the controlled release of vesicles containing acetylcholine (ACh) from the motor nerve terminal in response to the arrival of a nerve action potential. ACh crosses the synaptic cleft and binds to clustered acetylcholine receptors (AChR) on a specialised area of the muscle fibre, the NMJ. Activated AChR allows current to enter the muscle fibre and generate a localised membrane depolarisation which in turn activates voltage-activated sodium channels which propagates a muscle fibre action potential leading to muscle contraction.

Over many decades the use of animal models to manipulate this structure and probe its constituents has elucidated many aspects of its formation and function, although some questions remain. This review will highlight the breadth of our understanding that has come from the use of animal models.

The reproducible and robust nature of the NMJ signal transduction is a product of its precise structure and molecular organisation. The precise localisation of key components within the synapse and the characteristic post-synaptic muscle membrane invaginations are vital for its function. Indeed, this structure has evolved to be over-engineered to such a degree that there is a so-called “safety factor” that produces a post-synaptic signal that is more than required to generate a muscle action potential, thus allowing some loss of function before a catastrophic failure. It is disturbance of this safety factor that leads to variable degrees of signal failure, especially under rapid or repeated use, that underlies the various manifestations of NMJ disruption and fatigue. Animal models have been instrumental in investigating this safety factor disruption and testing modifying therapies to reverse it.

In this review I will describe how our understanding of the complex mechanisms underpinning neuromuscular transmission and NMJ structure has benefited from the use of animal models. Perhaps the ultimate animal model are humans, in the form of “experiments of nature”, have allowed key elements in the function and organisation of the NMJ to be pinpointed. These “experiments of nature” have resulted in a varied grouping of inherited syndromes all characterised by fatigable muscle weakness called congenital myasthenic syndromes (CMS). The content of this review is led by the discoveries of the mutant proteins responsible for these forms of neurotransmission disruption. Currently around 30 genes have been identified that harbour pathogenic mutations responsible for CMS [1,2]. For many forms of CMS animal models have helped to extend our understanding and investigate the role of each protein in NMJ structure and function, see Table 1. In the era before whole genome/exome screening clues to identify specific protein involvement in NMJ structure and/or function often came from animal knockout experiments or detailed study of CMS patients.

## 2. Methodology

**Figure 1:**
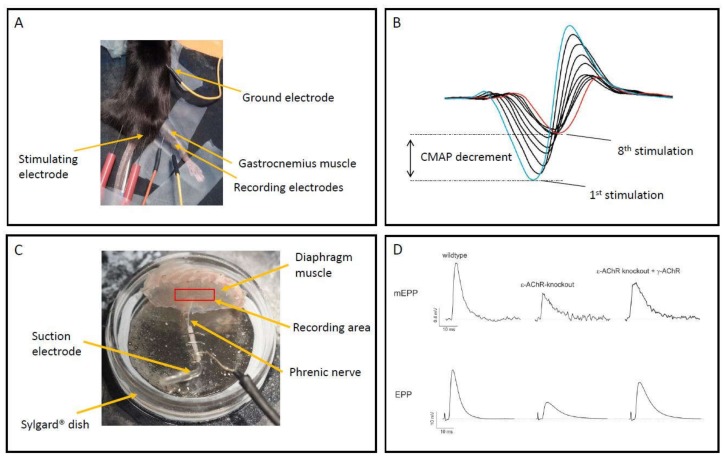
Panel (**A**) shows experimental setup for in-vivo electromyography of anaesthetised mouse, with location of stimulating and recording mono-polar needle electrodes. Panel (**B**) shows example trace of compound muscle action potential (CMAP) recorded for gastrocnemius muscle, significant decrement is evident between 1st and 8th stimulation at 10 Hz. Panel (**C**) shows experimental setup for sharp electrode recording from *ex-vivo* mouse phrenic nerve/hemi-diaphragm muscle, central area surrounding phrenic nerve branch within muscle where recordings are acquired is indicated. Panel (**D**) shows examples of miniature endplate potentials (mEPPs) and stimulated endplate potentials (EPPs) recorded from an 8-week-old wildtype mouse, an ε-AChR knockout mouse and an ε-AChR knockout mouse with human γ-AChR knocked-in. ε-AChR knockout mice have severely reduced mEPP and EPP amplitude due to diminishing post-natal expression of γ-AChR containing receptors with no adult AChR expression, knock-in of human γ-AChR partially restores mEPP and EPP amplitude and is a model for AChR-deficiency CMS.

Many different animals have been utilised to study the precise organisation and function of the NMJ, including *C. elegans*, *Drosophila* and zebrafish, but the mouse model has been the most informative and will be the prime focus of this review. The ultimate function of the NMJ is to produce a post-synaptic depolarisation, due to current passage through AChR positioned on the crests of the post-synaptic folds, such that voltage-gated sodium channels, resident in the depths of the post-synaptic folds, are activated and generate a propagating muscle action potential leading to contraction. Failure of this process can have many origins and animal models can be extremely useful in dissecting which is responsible. The mouse NMJ lends itself to study in this situation due to its large size and accessibility, facilitating microscopic study by immunofluorescence histology and functional analysis by electrophysiological methodologies. See Figure 1 for examples of methodologies for electrophysiological recording of neurotransmission in mouse models of CMS.

Localisation and density of pre-, post- and synaptic proteins can be visualised by use of specific antibodies and their rearrangement or loss in disease states determined. Fluorescently tagged proteins can be monitored in vivo and elucidate dynamic alterations and developing disease processes. Electron microscopy can provide an even more detailed examination of alterations of structure and/or localisation of synaptic proteins in disease states or following interventions.

Methodologies for the electrophysiological characterisation of animal models of NMJ dysfunction, in relation to models of myasthenia gravis, were recently described in detail [17]. Functional readouts of neuromuscular transmission failure can be obtained in vivo from electromyography of an anaesthetised animal, where recording electrodes are placed on or within a given muscle and compound muscle action potentials (CMAP) recorded following stimulation of the controlling motor nerve. This allows the assessment of signal transduction efficiency, how many muscle fibres within a muscle are recruited for a given motor nerve stimulation. If a given muscle fibre is not recruited, this indicates the post-synaptic depolarisation required to trigger voltage-gated sodium channels was insufficient. If the sodium channels are not triggered, no action potential will be generated in that fibre and it will not contribute to CMAP amplitude. Decrement in CMAP amplitude during repeated stimulation is a measure of fatigue, indicating that the safety factor has been increasingly compromised for individual muscle fibres which therefore no longer contribute to the CMAP and underlie the decrement in CMAP amplitude. These investigations can be repeated on individual mice allowing assessment of disease progression and treatment efficacy.

More detailed information about signal transmission can be obtained from *ex-vivo* nerve/muscle preparations, classically the diaphragm/phrenic nerve combination. With the insertion of microelectrodes into the muscle fibre, close to the NMJ, membrane potential changes or currents can be recorded. These are the result of AChR activation following release of pre-synaptic vesicles containing ACh, either spontaneously (miniature endplate potentials, mEPP) or following motor nerve stimulation (endplate potentials, EPP), the ratio of mEPP amplitude to EPP amplitude indicates how many vesicles were released following nerve stimulation and is referred to as the quantal content (QC). From these readouts many aspects of neurotransmission can be determined and changes to these readouts can specify which aspect of neurotransmission is distorted.

An important benefit of using animal models to investigate the NMJ is the ability to study functionality of the entire system by means of subjective clinical assessment of disease severity or objectively by measurement of muscle strength and fatigue. Muscular strength can be measured in isolated muscles and anaesthetised animals with transducers to measure strength or in free moving animals with weight lifting tasks. Fatigue is a crucial measure of progressive failure to achieve muscle fibre recruitment due to loss of safety factor; this can be measured with a grip test or inverted screen-hang test [18]. Usefully these measurements can be repeated during phenotypic development and following treatment.

The importance of functional measurement of animal models cannot be underestimated. The NMJ can appear disrupted but function close to normal [19], or the NMJ may appear macroscopically normal but this obscures abnormal function [20]. Thus, it is important and provides invaluable information about disease mechanisms if animal models are explored to their maximum.

## 3. Animal Models Used in the Study of CMS-Associated Proteins

### 3.1. Post-Synaptic

#### 3.1.1. AChR Deficiency

**Figure 2:**
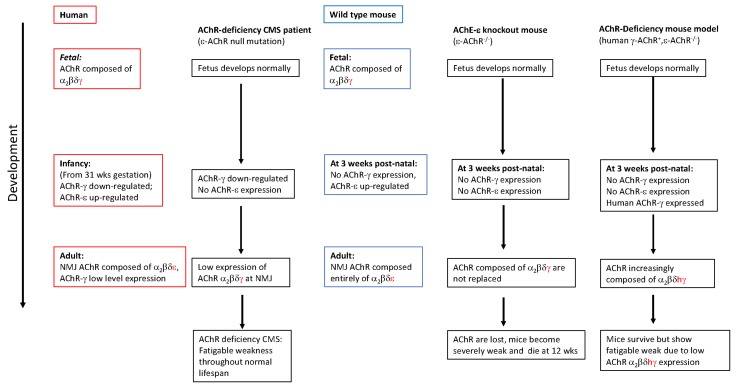
Diagrammatic representation of the developmental changes in AChR composition in humans and mice. Differences between human and mouse expression of ε- and γ-AChR are highlighted and consequences for AChR-deficiency CMS patients and mouse models of AChR-deficiency are compared. Mouse model expressing human γ-AChR in ε-AChR knockout background provides robust model of human CMS phenotype.

Many of the proteins that harbour CMS mutations are responsible for the specific localisation and concentration of the AChR at the NMJ. Indeed, to ensure robust and repeatable neurotransmission AChR must be highly concentrated at the peaks of post-junctional folds at a concentration of ~9000 per μm^2^ [21]. Failure to achieve or maintain this acute localisation leads to failure of neurotransmission and fatigable muscle weakness. Mutations of the gene encoding the AChR ε-subunit that underlie AChR deficiency give rise to the most common subgroup of CMS patients [22,23]. These mutations can either prematurely truncate one of the AChR subunits or lead to complete or partial failure of AChR subunit assembly. In normal development embryonic muscle AChRs contain α, β, δ and γ subunits. At around the time of birth a developmental switch occurs which reduces the expression of γ-subunit and turns on the expression by sub-synaptic myonuclei of the ε-subunit [24,25]. The ε-subunit then replaces the γ-subunit within the AChR pentamer at synaptic sites. This subunit switch generates adult AChR channels with different properties that are better adapted to its functional role at the adult NMJ. The consequences of an inability to perform this switch have been investigated using mouse models with the ε-subunit ablated [26,27]. Epsilon null mice are born indistinguishable from normal litter mates but subsequently develop a distinct phenotype with slowed growth and loss of grip strength leading ultimately to overt weakness and death at ~3 months. Endplate currents show lack of transition from fetal AChR kinetics to adult kinetics demonstrated by slower decay time constants typical of fetal AChR burst characteristics, a transition that normally occurs between postnatal day 5 (P5) and P30. Amplitude of endplate currents is also reduced from P5 to P30 and P70 indicating loss of post-synaptic receptor number and density; by P70 null mice have only 5% of control AChR. Loss of AChR is also associated with loss of post-synaptic membrane folding and fragmentation of the NMJ. Interestingly, in muscle biopsy samples from CMS patients with ε-subunit null mutations, AChR measured by α-bungarotoxin binding is 10–30% of controls. The lethality of ε-subunit knockout in mice within 3 months of birth contrasts with CMS patients with ε-subunit null mutations; these patients often show mild fatigable muscle weakness with no limitation of lifespan. Analysis of AChR subunit mRNA in patient muscle biopsies revealed a low but persistent expression of γ-subunit mRNA even in adult muscle [28]. By contrast, in mice, γ-subunit expression is barely detectable by postnatal week 3, in γ AChR knockout mice expression persists for longer, but insufficient expression for survival beyond postnatal week 12. It has been postulated that this low-level expression in CMS patients is sufficient to sustain limited neurotransmission for survival but not eliminate fatigable weakness. This scenario was examined by expressing human AChR γ-subunit driven by a muscle specific promoter in the ε-subunit knockout mice [3]. Epsilon-knockout mice with expression of human γ-AChR displayed a phenotype with greater similarity to the human CMS phenotype, summarised in Figure 2. These mice were weak and small compared to normal littermates, but they did not deteriorate with aging and amplitude of mEPPs and EPPs was significantly enhanced compared to ε-knockout mice. Staining of NMJ revealed a disrupted pretzel structure. This model demonstrates that lifelong expression of γ-subunit can substitute for loss of the ε-subunit and facilitate surface expression of AChR that can sustain life, even if that AChR is of the fetal subtype. Importantly, humans and mice differ in the level of continued expression of AChR-γ subunit. In humans, expression of γ-subunit, sufficient for at least some NMJ function, is sustained throughout life; whilst in mice its expression is restricted to early development. This provides an explanation for why AChR deficiency CMS due to mutations in the ε-subunit are relatively prevalent. Thus, here, a refined model that closely replicates the human disease phenotype has provided a much better tool for understanding the disease mechanism and a better resource for testing therapeutic interventions.

#### 3.1.2. AChR Kinetic Mutations

Once accumulated at the post-synaptic membrane, the precise way AChR respond to binding ACh is fundamental to the physiological process of neurotransmission. Many CMS mutations in the subunits of AChR can alter the way this ion channel opens and closes with direct and indirect consequences for neurotransmission. Mutant AChR function can be studied in heterologous expression systems and their precise kinetic alterations characterised. These kinetic mutations result in 3 possible outcomes; either the ion channel remains open for too long (Slow Channel CMS (SCCMS)); the openings are too brief (Fast Channel CMS) or conductance of the open channel is reduced (found is one case [29]). For fast channel CMS the pathological mechanism is relatively uncomplicated, in this situation activation of mutant AChR produces muscle membrane depolarisation that is insufficient to activate voltage-gated sodium channels with the resultant failure to instigate muscle action potentials and contraction, similar to AChR deficiency. However, for SCCMS the pathological mechanism is not as well understood and several animal models for SCCMS have been developed that have incorporated mutant AChR with slow channel kinetic alterations into the synapse. These models have been used to elucidate disease mechanisms and test therapies.

**Figure 3:**
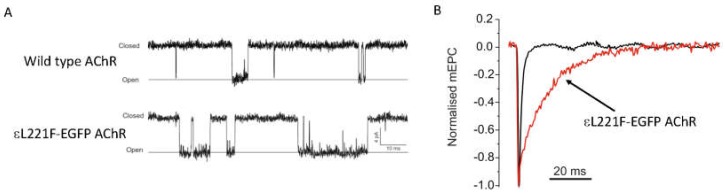
Panel (**A**) shows single channel recordings from HEK 293 cells transfected with either wild type AChR subunits or wild type AChR subunits plus εL221F-EGFP mutant AChR subunit. Prolonged bursts of openings are evident (downward deflections) stimulated by low concentration of ACh (100 nM). Panel (**B**) shows mEPC recordings from diaphragm muscle of mutant mice expressing εL221F-EGFP containing AChR (red trace). Endplate currents are prolonged in these mice as a result of the increased duration of AChR burst kinetics.

Since 1996, seven mouse models of SCCMS have been developed and characterised and have been used to investigate the effects of patient SCCMS mutations or mutations engineered to prolong AChR open duration. In most cases overexpression of the mutant AChR subunit was utilised [4,5,6,7]. In other models, homologous recombination was used to insert the point mutation (εL221F, AChR mutants are referred to by their legacy numbering where residue +1 is the first codon of the mature peptide) into the native AChR subunit [8], or transgene expression of AChR containing the εL221F mutation in a mouse with wildtype AChR ε-subunit knocked out [9]. See Figure 3 for example of SCCMS kinetic alterations as a result of insertion of mutant ε-AChR (εL221F-EGFP AChR). Each of these models have successfully recapitulated the key neurotransmission pathogenic characteristic observed in SCCMS patients, namely a prolonged endplate current. However, how this ultimately leads to fatigable weakness in all cases is unclear. When a panel of SCCMS mouse models were directly compared, the degree of muscle weakness was most related to the extent of prolongation of AChR open duration, relative calcium influx and muscle activity, adding credence to the hypothesis that detrimental sequelae from excess sub-synaptic calcium are prominent in the pathological mechanisms underlying SCCMS. Indeed, in the weakest mice more markers of calcium overload were observed, such as localised calcium accumulation, calcium-dependant activation of caspases or myopathic changes. These effects could be reversed by denervation thus directly linking cholinergic activity with calcium overload induced damage [10]. Furthermore, activation of calpain (another calcium-activated protease) is observed in the εL269F SCCMS model [11]. This is associated with calcium accumulation and activation is reduced by axotomy or AChR blockade. Of note, calpain may play an intrinsic role in the stability of AChR clusters. Calpain is associated with rapsyn in an agrin dependent manner. Rapsyn acts to inhibit calpain, but once activated by increased intracellular calcium it cleaves p35 to p25 and leads to AChR cluster dispersal via cdk5 activation [30]. Therefore, mutant AChR activity might exacerbate this pathway and lead to excessive AChR dispersal. Overexpression of calpastatin in these SCCMS mice, which inhibits calpain, leads to improved muscle strength and neurotransmission. However, other calcium-activated processes are still active, and thus not all detrimental aspects of the mutant AChR are prevented.

Mouse models of SCCMS have also demonstrated the efficacy of blockade of mutant AChR by fluoxetine [12] (successfully used to treat SCCMS patients [31]) and the relatively limited response to ephedrine [9].

Although calcium overload and its detrimental consequences may be the dominant pathological mechanism a SCCMS mouse model (overexpressing δS262T) highlighted the potential importance of an additional pathogenic mechanism [20]. Although endplate current (EPC) was prolonged there were no changes to the NMJ structure as seen in other models of SCCMS. The mice were not weak, but EPC amplitude was reduced in addition to the predicted prolongation and occurred even though AChR numbers were not reduced and no endplate myopathy was detected. Upon high frequency repetitive nerve stimulation EPC current amplitude was more severely reduced compared to control mice. The reduction in EPC amplitude is likely the result of entrapment of mutant AChR in a non-conductive desensitised state, a mechanism was postulated by Milone et al. in 1997 when kinetic modelling of a CMS mutation αV249F showed enhanced steady-state desensitisation in addition to prolonged channel openings [32]. Wild type AChR do not usually enter the desensitized state since ACh removal is so rapid, however alterations to kinetic behaviour due to the point mutations can not only prolong ACh induced open duration but can also increases the chance of entering a desensitised state. If time between stimulations is insufficient for the channel to leave the desensitised state an increasing proportion of the available channels will be non-conductive and consequently reduce the overall EPC amplitude. It would increase the danger of failing to reach threshold for activation of synaptic sodium channels. The same phenomenon was also observed in SCCMS mouse models incorporating the εL221F mutation [8,9]. Furthermore, this mechanism would act synergistically with endplate myopathy and loss of AChR (in those cases where calcium overload induced myopathy does occur) to further endanger threshold crossing.

#### 3.1.3. MuSK/LRP4

Agrin is a nerve-derived factor that is essential for development of the NMJ. The muscle membrane receptors for agrin were established using targeted knock-out of candidate proteins in mice. MuSK (Muscle-Specific Kinase) was identified as the crucial component of the response to agrin that coordinates localised specialisation of the muscle membrane. MuSK is a muscle derived tyrosine kinase and disruption of the *MUSK* gene in mice proved lethal causing a failure of NMJ formation (with post-synaptic and synaptic proteins absent) and excessive neurite outgrowth [33]. In myotubes, agrin fails to induce AChR clustering if MuSK is not present [34]. However, agrin does not directly bind to MuSK and is not the agrin receptor. It was subsequently shown that, although MuSK is required, LRP4 (Low Density Lipoprotein Receptor (LDLR)-related protein 4) was the actual receptor for agrin, in a complex with MuSK. Mice with genetic disruption of the *LRP4* gene show a remarkably similar phenotype to the MuSK knockout mice. [35]. Application of agrin leads to the formation of an LRP4-MuSK-DOK7 complex, MuSK auto-phosphorylation and AChR clustering in myotubes [36,37]. Thus, activation of the Agrin-MuSK-LRP4-DOK7 complex coordinates the localised pre- and post-synaptic specialisation forming the NMJ and is required for its maintenance. Additionally, MuSK can act via an agrin-independent pathway that prepares the muscle membrane prior to the agrin signal and directs the localisation of the NMJ to the central zone of muscle fibres [38,39,40,41]. Furthermore, it has been postulated that Wnt signally molecules also interact directly with the cysteine-rich-domain of MuSK and are involved in NMJ pre-patterning independent of neural agrin [42,43,44].

Mutations in *MUSK* have been identified in a small number of CMS patients. One CMS patient mutation (p.V790M) has been incorporated into a mouse model (the analogous mouse is p.V789M) to enable a more detailed examination of the consequences of this alteration in MuSK function [13]. The homozygous mutant mouse (MuSK^V789M/V789M^) showed no phenotype. Only when the V789M mutant was hemizygous with the MuSK knockout (MuSK^V789M/-^) was an overt phenotype observed. This mouse was under-weight, weaker with skeletal deformations and had reduced survival. Muscle contractile performance was reduced, and tetanic contraction was not well maintained. In diaphragm muscle, the frequency of mEPPs was reduced although amplitude was maintained. However quantal content was reduced with a concomitant reduction in EPP amplitude. Structurally motor nerve outgrowth extended outside the normal endplate zone and NMJs were fewer and smaller and deteriorated with age. Interestingly, changes also occurred in expression of key NMJ proteins, notably MuSK, AChR α and γ subunits mRNA were upregulated whilst AChR ε-subunit mRNA was reduced, suggesting that MuSK plays a role in regulation of gene expression of other NMJ proteins. Some features of the MuSK^V789M/-^ mouse are similar to MuSK-CMS patients and may provide a useful tool for further understanding of this CMS and offer the possibility for the testing of therapeutics.

#### 3.1.4. Rapsyn

For the NMJ to function correctly AChR must be arranged at high density on the peak of the post-junctional folds, rapsyn is the structural protein that is vital for this organisation. Rapsyn was initially identified as a 43KD protein isolated from torpedo electric organ and skeletal muscle that enabled highly dense clustering of AChR at the NMJ [45,46]. In vivo evidence for this role was provided by knock-out experiments [47], producing neonatal lethality in homozygous pups with a typical lack of AChR cluster formation and excessive branching of the motor nerve. However, due to lethality more subtle roles of rapsyn were difficult to establish. Subsequently, mutations were identified in CMS patients. In contrast to the knock-out mice, patients with *RAPSN* mutations can have a varied phenotype, often quite mild. These CMS mutations have highlighted several important functional domains within the rapsyn molecule [48,49], that can interfere with its self-aggregation, interaction with actin or association with AChR subunits and ultimately disrupt AChR clustering. Additionally, a mutation has been identified in the AChR δ subunit that interferes with the rapsyn-AChR interaction, which disrupts AChR clustering in myotubes [50]. More recently an additional enzymatic function of rapsyn has been postulated [14]. Mouse models were generated to investigate this enzymatic function of the RING-domain of rapsyn (C366A). This point mutation was shown to inhibit E3-ligase activity of rapsyn, a known property of RING-H2 domains. The role of the RING-domain E3-ligase activity was then examined by rescue of a lethal rapsyn knock-out mouse by introduction of mutant rapsyn (C366A) or by using CRISPR-technology to knock-in the C366A missense mutation. In both cases expression of the mutant rapsyn lead to perinatal lethality with lack of AChR clusters and extensive motor nerve arborisations. This was not the result of protein instability, loss of its interaction with actin, lack of accumulation at the NMJ or interference of its interaction with the AChR subunits. However, this point mutation did disrupt the ability to stimulate the formation of AChR clusters in a heterologous cell system. Further investigation revealed that the RING-domain E3-ligase activity catalyses the neddylation of the AChR δ subunit (at position K397), and inhibition of this process disrupts the Agrin-MuSK-DOK7-Rapsyn-AChR clustering pathway. Thus, the E3-ligase activity is a function of rapsyn separate from its role as a scaffold protein but is vital for NMJ structural organisation, as demonstrated in two animal models.

#### 3.1.5. DOK7

The role of DOK7 (Downstream of Tyrosine Kinase-7) in post-synaptic specialisation of the muscle membrane, as part of the agrin-MuSK-AChR clustering pathway was first shown by elaborate in vitro experiments examining the dependence of clustering on the interaction of DOK7 with MuSK in myotube and heterologous expression in HEK 293 cells. Proof of its vital role was shown by knock-out experiments in mice. *DOK7* knock-out mice produced only non-viable pups with severely disorganised NMJ, with the typical lack of AChR clustering and exaggerated span of the motor nerve arborisation [51]. These findings identified a novel potential target for pathogenic CMS mutations and subsequently many mutations were found in *DOK7* [52] leading to the classification of a new clinical subtype of CMS [53]. The lethal nature of *DOK7* knock-out mice renders their utility to further study the in vivo role of DOK7 limited. Homologous recombination was used to generate the equivalent of the human *DOK7* CMS mutation (c.1124_1127dupTGCC) in a transgenic mouse line. Mice were viable for up to 2 weeks, thus allowing the trial of interventions that could rectify the NMJ disorganisation to prolong their lifespan [15]. These mice, although viable for ~2 weeks are much more severely affected than the CMS patients that carry the same mutation. This animal model was used to test the potential for DOK7 upregulation to restore NMJ dysfunction. Injection at P9 of adeno-associated virus (AAV) encoding mouse DOK7 was able to generate oversized pre- and post-synaptic specialisations and substantively restored lifespan and muscle strength. The same AAV derived DOK7 was also able to restore function in other animal models with defective NMJ, Emery-Dreifuss muscular dystrophy [15] and amyotrophic lateral sclerosis [54]. Electrophysiological measurement of functional neurotransmission was not reported for these animal models, so precisely how the enlarged synapses function is not known. DOK7 also has a function downstream of its interaction with MuSK, these were investigated using another animal model [55]. Via its C-domain DOK7 interacts with CrK and CrK-L, the importance of this was demonstrated in vivo using a muscle specific knock-out. This model produced non-viable offspring, but E18.5 embryos had disorganised motor nerve distribution with fewer and smaller synapses, suggesting lack of innervation and/or denervation resultant from CrK and Crk-L knockout. The AChR β-subunit was phosphorylated, suggesting that the DOK7-MuSK-AChR signalling pathway was still intact.

### 3.2. Synaptic

#### 3.2.1. ColQ

Within the synapse, removal of ACh is governed by its enzymatic destruction catalysed by acetylcholine esterase (AChE) [56]. It is this fast process that terminates the ACh signal in preparation for the next impulse. The precise location and concentration of this enzyme is critical to efficient neurotransmission. AChE exists in many forms, but the so-called “asymmetric” form is preferentially located at the NMJ. This is achieved by tethering of AChE to the basal lamina by collagen Q (ColQ) which has C-terminal binding sites for perlecan, a heparan sulphate proteoglycan constituent of the basal lamina [57] and can bind up to 12 molecules of AChE via its proline-rich attachment domain (PRAD) towards the N-terminal [58]. Mutations have been found in *COLQ* that underlie a form of CMS, originally described as endplate AChE deficiency. Many of these mutations either prevent the association of AChE with ColQ or prevent the binding of ColQ to the basal lamina of the NMJ [59,60].

Intricacies for the role of ColQ-AChE in neuromuscular transmission were revealed by a *COLQ* knockout mouse model [61]. AChE was completely absent from the NMJ in these mice, by histochemistry and immunostaining, confirming the obligatory role of ColQ in localising AChE to the NMJ. Surprisingly, given the complete absence of neuromuscular AChE, the phenotype was relatively mild. Homozygous *COLQ* knockout pups were initially indistinguishable from normal littermates. Muscle tremor upon moving was observed from P5 onwards, they developed more slowly than littermates with 50% mortality by P21, but ~20% lived past 3 months. Electrophysiological recordings of muscle fibre mEPPs showed prolongation compared to wildtype, but no further prolongation following blockade of residual AChE by fasciculin; indicating no functional AChE in the NMJ. Furthermore, in wild type controls fasciculin greatly prolonged the mEPP duration and caused flaccid paralysis when injected into mice but had only mild effects on knockout mice. These data suggest the knockout mice have adapted to lack of AChE, with structural evidence of nerve terminal encasement by Schwann cells reducing ACh release. In these conditions slower breakdown of ACh may also be accomplished by butylcholinesterases located on terminal Schwann cells [62].

The same *COLQ* knockout mouse model was furthered studied, ColQ interaction with MuSK and other components of the NMJ that were not a focus of the initial study [63]. It was shown that lack of ColQ, possibly via a lost interaction with MuSK, changed the pattern of mRNA expression of many NMJ and ECM proteins. All 5 muscle AChR subunits mRNAs were upregulated, and expression of fetal AChR (α_2_βδγ) was visually and functionally detectable in mature (P30) diaphragm muscle at a developmental stage where fetal receptors are not normally found. Other elements of the basal lamina and post-synaptic machinery were also upregulated, including AChE, MuSK, DOK7, nidogen 2, syntrophin β2 and perlecan whilst rapsyn was downregulated. These data suggest the ColQ may play a role in gene expression critical for NMJ development and homeostasis in addition to tethering AChE and ACh removal. The control of gene expression by ColQ may explain the relative complexity of the AChE deficiency CMS phenotype, however, the mechanisms of these interactions is still to be elucidated.

#### 3.2.2. Agrin

The recognition of Agrin (*AGRN*) as a vital nerve-derived organising factor responsible for the localisation and organisation of the NMJ is one example of how animal models have provided definitive evidence of the role of NMJ proteins in health and disease. Agrin was first identified as one of a number of factors that could aggregate muscle AChR in a myotube in vitro system [64], originally isolated from the insoluble extract of torpedo electric organs [65]. Homozygous knock-out of the neural-specific isoform of agrin in a mouse model resulted in a marked reduction in post-synaptic AChR aggregates (in number, size and density) and additionally abnormal intramuscular nerve branching and pre-synaptic differentiation, resulting in death at birth [66]. Further evidence of its role (and utility of animal models) was provided by study of mutant agrin. A missense mutation in *AGRN* (p.Gly1709Arg) derived from a CMS patient was confirmed as pathogenic only following injection of recombinant wildtype or mutant agrin into rat soleus muscle; the mutant protein causing destabilisation of NMJ structure [67]. Knock-out models are not always the most informative of disease processes since often they result in neonatal lethality. An ENU-generated point mutation of *AGRN* (p.Phe1061Ser) produced homozygous mutant mice with a definitive phenotype but life span of up to a few months in C57BL/6J background, of note, in a different genetic background the effects were milder and lifespan markedly increased [16]. This point mutation elicits a phenotype, not evident until P13, of progressive degeneration of pre- and post-synaptic architecture with evidence of motor nerve sprouting and synaptic AChE loss. The phenotype is similar to that generated by CMS missense mutation (p.Val1727Phe) co-inherited with the null mutant (p.Q353X) where small fragmented NMJ were observed [68]. This mutation disrupted in vitro aggregation of AChR whilst for the two previous mutations in vitro functionality was unaffected, suggesting apparent different functional domains of agrin.

#### 3.2.3. The Extracellular Matrix (ECM)

Many proteins are secreted from both muscle fibre and motor nerve to populate the space around the NMJ and in the synaptic cleft, they accumulate to form the extracellular matrix and play a crucial role in the development and maintenance of the NMJ. Knowledge of the full role of the ECM is still accumulating and is not limited to the NMJ [69]. Recently, it addition to ColQ and Agrin, two more ECM components have been implicated in CMS and their roles elucidated by use of animal models; these are Laminin β2 and Col13A1. Additionally, the roles of other ECM components on NMJ development, structure and function have been studied in animal models; these include integrin α3 and Col IV.

Mutations in *LAMB2* leading to truncation of laminin β2 have been associated with the oculorenal syndrome, Pierson syndrome [70]. Ablation of *LAMB2* in mice also demonstrated a neuromuscular phenotype [71]. Subsequently mutations in *LAMB2* were reported in a form of CMS associated with congenital nephrosis and ocular malformations [72]. In this patient the neuromuscular disruption closely resembles observations from the knock-out mice. In both the ultrastructure of the NMJ was affected, with reduced active zone and presynaptic vesicles, Schwann cell encasement and reduced post-junctional folding. From electrophysiological recordings, both had reduced mEPP frequency and reduced quantal content. Thus, structural defects resulting from loss of laminin β2 reduce functionality of the NMJ, although this could be secondary to kidney failure. The patient underwent kidney transplantation at 15 months which enabled survival and allowed her motor deficit to become apparent.

Collagen XIII is a non-fibrillary collagen with a short intracellular domain, single transmembrane domain and a large extracellular collagenous ectodomain that is expressed in muscle. The ectodomain of this protein can be proteolytically shed into the synaptic cleft and forms part of the ECM [73]. Its role in NMJ development and maintenance was investigated with two mouse models where the alpha 1 chain (COL13A1) of the collagen was disrupted; one where COL13A1 was knocked out (Col13A1^−/−^); and one where shedding of the ectodomain was impaired (Col13A1^tm/tm^) [73,74]. Col13A1^−/−^ mice were smaller and weaker than normal littermates but lifespan was unaffected. Electrophysiologically these mice had less spontaneous activity, reduced quantal content and a reduced readily releasable pool of vesicles. Ultrastructural investigation revealed Col13A1^−/−^ mice to have incomplete pre- and post-synaptic adhesion and aberrant Schwann cell migration into the synaptic cleft with reduced surface contact for neurotransmission, consistent with the electrophysiological data. These defects are the result of impaired interaction of Col13A1 with the ColQ tail of the AChE structure. Mice with impaired Col13A1 shedding (Col13A1^tm/tm^) had largely similar defects, suggesting that the most important functions of Col13A1 are mediated by the transmembrane form and not the shed ectodomain. Truncation mutations in *COL13A1* have recently been identified in myasthenic patients and classified as a novel form of CMS (CMS 19). The patient phenotype and response to treatment is consistent with the role of COL13A1 in NMJ development and maintenance [75].

The complex in vivo role of three gene families (Fibroblast Growth Factor (FGF), laminin, and collagen IV) that generate constituents of the ECM have been assessed using various mutant mice; these families of proteins had all shown similar actions on cultured motor neurons [76]. It was found that each of these factors had distinct and sequential actions on NMJ development. FGFs and collagen α1/2(IV) chains direct the initial differentiation of nerve terminals, β2 laminins promote their maturation, and collagen α3–6 (IV) chains are required to maintain the NMJ. These roles are temporally organised by developmental regulation of expression.

A principle receptor of the laminins and collagens are the integrin family of molecules [69], recently the role of the presynaptic receptor integrin α3 was examined in mice with partial or complete ablation [77]. These mice had been examined previously. Knockout of integrin a3 was associated with kidney and lung organogenesis, but its role in NMJ structure and function was not examined [78]. Homozygous mice (Itga3^−/−^) show perinatal lethality but heterozygous (Itga3^+/−^) mice have no overt phenotype but reveal subtle structural and functional changes. The combination of ultrastructural microscopy, immunostaining and electrophysiology of Itga3^−/−^ and Itga3^+/−^ mice showed that integrin α3 has several roles at the NMJ. It localises elements of the presynaptic active zone and facilitated efficient vesicular release whilst also regulating structural integrity. Loss of integrin α3 was associated with progressive structural changes and autophagy-features of ageing muscle, with examples of nerve terminal detachment, suggesting a role in physical anchorage of pre- and post-synaptic elements of the NMJ. Human mutations of *ITGA3*, which encodes integrin α3, leading to total loss of integrin α3 expression are associated with severe kidney and lung malformation and skin fragility; suggesting a multi-organ role [79] as seen in the itga3^−/−^ mice. Mild muscle hypertonia was reported as only a minor feature, but since these children died at only a few months of age, perhaps NMJ disruption might become apparent with integrin α3 mutations that alter rather than ablate function and thus allow longer term effects on NMJ structure to be revealed.

### 3.3. Pre-Synaptic

The use of animal models to investigate pre-synaptic CMS mechanisms is not extensive, perhaps complicated by shared machinery at the motor nerve terminal and central synapses and the relatively recent discovery of many of the causative gene mutations. Currently seven genes have been identified in CMS patients that code for proteins involved in process related to ACh recycling and vesicular release; (*CHAT*, *SLC5A7* and *SLC18A3*) and (*SYT2*, *VAMP1*, *SNAP25* and *UNC13A*) respectively; and often underlie severe CMSs often with episodes of apnoea and extensive co-morbidities related to CNS sites of action. Of note, *CHAT*, *SLC5A7* and *SLC18A3* mutations are associated with ACh recycling and the phenotype can be mild, whereas *VAMP1*, *SNAP25B* and *UNC13A1* are involved in neurotransmitter release and exocytosis mechanisms common to many synapses and a multisystem severe phenotype including myasthenia is expected. This group of CMS-related pre-synaptic targets have recently been the subject of review [2] and since animal models examining the role of these proteins at the NMJ in the aetiology of CMS have not been reported (except 3) I will not focus in detail on this area of the NMJ.

Animal models of NMJ disruption have been studied for *CHAT* and *SYT2*; *CHAT* encodes the protein choline acetyltransferase (ChAT) and is responsible for biosynthesis of ACh from choline and acetyl CoA, whilst *SYT2* encodes synaptotagmin-2 which is the calcium sensor for fast vesicular release from the motor nerve terminal. Electrophysiological signature of CMS due to *CHAT* mutations is a decrementing CMAP that is slow to recover and affected patients suffering fatigable weakness with severe and sometimes fatal apnoeic attacks [80]. Mutations that have been identified can affect ChAT expression and kinetic activity. Two mouse models of *CHAT* knockout exist and show similar effects [81,82]. In both cases knockout is lethal with severe disruption of motor nerve targeting and post-synaptic maturation. No neurotransmission is detected (both spontaneous and stimulated) but functional clustered AChR are present on the muscle fibre, with many fibres multi-innervated. These findings support the hypothesis that cholinergic activity itself plays a role in NMJ targeting, NMJ pruning and maturation as well as neurotransmission.

CMS due to mutations in *SYT2* are characterised by dominant inheritance, a Lambert-Eaton like neurophysiology (post-exercise CMAP amplitude facilitation), foot deformities and fatigable muscle weakness [83]. The patients were reported to respond well to 3–4 diaminopyridine [84] which prolongs nerve terminal depolarisation and maximises vesicular release. However, these patients mostly have a hereditary neuropathy, with only some showing myasthenic features. A *Drosophila* model incorporating the homologous mutation in synatotagmin-2 from a CMS patient (P308L) mutation has been recently studied [85]. This mutation disrupts the C2B Ca^2+^-binding pocket of synaptotagmin-2. In *Drosophila* this mutation is lethal when homozygous, in the heterozygous condition (as with the CMS patient) disruption of neurotransmission is evident. The equivalent of EPP in the mutant *Drosophila* was significantly reduced whilst mEPP frequency and amplitude were unaffected. More detailed examination revealed a reduction in calcium sensitivity for vesicular release but an increase in facilitation following high frequency stimulation. Behaviourally the mutant flies were less active and fatigued more readily; this was accompanied by a reduced lifespan. These observations confirm the pathogenicity of the mutations found in *SYT2* of this rare form of CMS and indicates the mechanism is via an alteration in calcium sensitivity of the vesicle release machinery.

Patients with either a homozygous truncation mutation or missense mutation of critical conserved residue in VAMP1 (vesicle-associated membrane protein 1) show muscle weakness, developmental delay and respond well to pyridostigmine. Lambert-Eaton-like electromyography was suggestive of a pre-synaptic site of action [86]. VAMP1 (a member of the synaptobrevin family) is part of the vesicular docking machinery. A spontaneously generated mouse model of a *VAMP1* mutant (*VAMP1*^lew/lew^) was previously described [87] that showed lethal wasting phenotype with reduced movement and death before weaning. A truncation mutation was identified in the coding region of *VAMP1* and expression of VAMP1 protein was undetectable. Subsequent neuromuscular electrophysiological examination of these mice found reduced EPP amplitude with facilitation upon repetitive stimulation, consistent with a pre-synaptic defect. NMJ were correctly located but significantly smaller compared with control mice [86].

## 4. Animal Models in Myasthenia Gravis

Prior to the appreciation of inherited forms of disrupted neurotransmission, understanding of the structure and function of the NMJ derived from the study of acquired forms of autoimmune myasthenia, myasthenia gravis (MG) and Lambert-Eaton myasthenia. In this field the use of animal models was crucial in understanding the pathophysiology of the assault on the NMJ. The target for ~85% of cases of myasthenia gravis as the AChR was established almost by accident when a rabbit immunised against AChR isolated from *Electrophorus electricus* electric organ; for the purposes of generating anti-AChR antibodies; itself became flaccid with abnormal electromyography, an effect reversed by AChE inhibition [88]. Since this time many animal models have been utilised to study the immunobiology and physiology of the acquired myasthenias (reviewed [89,90,91,92]). Animal models have also been vital in establishing the direct pathogenicity of other autoimmune antigens such as MuSK [93,94] and investigating the role of LRP4 and the potential pathogenicity of these autoantibodies [95,96,97].

A finding of particular note from comparisons of AChR-MG and MuSK-MG in animal models was the observation that homeostatic compensation occurs in AChR-MG (an increased quantal content is found when mEPP amplitude is decreased due to AChR loss [98]) whereas with MuSK-MG no increase in quantal content is observed and EPP amplitude is more severely reduced [93]. This difference might contribute to the increased severity and treatment refractoriness of MuSK-MG [99].

The ability of the neuromuscular synapse to adapt to changes in signal output resulting from perturbations such as autoimmune attack is another vital mechanism for the maintenance of transmission, termed homeostatic synaptic plasticity. The factors that control this adaptation are not fully understood and are the subject of an excellent recent review [100]. A recent particularly intriguing hypothesis is that the AChR itself acts as a trans-synaptic signalling molecule and the need for adaptation is not itself signalled by changes AChR current [101]. A fuller understanding of this and other mechanisms will provide new avenues for the modification of NMJ function that could be tested in animal models of NMJ dysregulation.

## 5. Conclusions

Provided the animal models reflect the *in-vivo* human biology, models of the neuromuscular junction they can be highly informative. The underlying pathway for NMJ synapse formation was uncovered from a series of knock-out mouse models. Animal models continue to be vital in understanding the role of many essential proteins responsible for the function and structural organization of the NMJ. Use of animal models have also been instrumental in revealing disease mechanisms in CMS and will continue to be vital as new CMS-causing mutations come to light. The mouse NMJ in many situations accurately reflects the human neuromuscular synapse in health and disease and provides a crucial tool to explore disease mechanisms and direct appropriate therapy. In many cases use of animal models has revealed the complex role of NMJ proteins in both structural and functional aspects than would not be apparent by isolated *in-vitro* assay alone. To elicit the maximum information from these models it is vital to fully investigate multiple aspects of the phenotype including the analysis of functional, biochemical and structural features.

## Figures and Tables

**Table 1:** Summary of specific CMS-related mutant animal models that replicate aspects of disease phenotype.

CMS Disorder	CMS Subtype	Gene	Description	References
AChR-Deficiency	CMS4C	*CHRNE*, *CHRNG*	Expression of γ-AChR in ε-AChR knockout background generates weak mice with reduced endplate depolarisation but normal lifespan.	[3]
AChR-Slow channel kinetic	CMS4A, CMS1A, CMS3A	*CHRNE*, *CHRNA1*, *CHRND*	Expression of slow channel kinetic mutant AChR replicates prolongation of AChR current, muscle weakness, calcium overload and response to treatment.	[4,5,6,7,8,9,10,11,12]
MuSK	CMS9	*MUSK*	Hemizygous expression of V789M mutant in knockout background generates overtly weak mouse with defects of NMJ structure and neurotransmission.	[13]
Rapsyn	CMS11	*RAPSN*	Mutation within RING-domain of rapsyn inhibits E3-ligase activity, disrupts AChR cluster formation, motor nerve targeting and is perinatally lethal.	[14]
DOK7	CMS10	*DOK7*	Duplication mutation (c.1124_1127dupTGCC) disrupts NMJ formation and is perinatally lethal.Overexpression of DOK7 rescues phenotype.	[15]
Agrin	CMS8	*AGRN*	Chemically generated missense mutation causes NMJ degradation with decreased AChR density and reduced lifespan.	[16]

## Abbreviations

AAV	Adeno-Associated Virus
ACh	Acetylcholine
AChE	Acetylcholine Esterase
AChR	Acetylcholine Receptor
ChAT	Choline Acetyltransferase
CMAP	Compound Muscle Action Potential
CMS	Congenital Myasthenic Syndrome
Col13A1	Collagen, Type XIII, α-1
Col-Q	Collagen Like Tail Subunit of Asymmetric Acetylcholinesterase
DOK7	Downstream of Tyrosine Kinase-7
EPC	Endplate Current
EPP	Endplate Potential
LRP4	Low Density Lipoprotein Receptor (LDLR)-related protein 4
mEPP	Miniature Endplate Potential
MuSK	Muscle-Specific Kinase
NMJ	Neuromuscular Junction
P	Postnatal day
QC	Quantal Content
Rapsyn	Receptor-Associated Protein of the Synapse
SCCMS	Slow Channel Congenital Myasthenic Syndrome
SCL5A7	Solute Carrier Family 5 (Choline Transporter), Member 7
SLC18A3	Solute Carrier Family 18 (Vesicular Acetylcholine), Member 3
SNAP25	Synaptosomal-Associated Protein, 25-KD
SYT2	Synaptotagmin 2
VAMP1	Vesicle-Associated Membrane Protein 1
